# Acute kidney injury predicts all‐cause mortality in patients with cancer

**DOI:** 10.1002/cam4.2140

**Published:** 2019-04-09

**Authors:** Eunjeong Kang, Minsu Park, Peong Gang Park, Namyong Park, Younglee Jung, U Kang, Hee Gyung Kang, Dong Ki Kim, Kook‐Hwan Oh, Kwon Wook Joo, Yon Su Kim, Hyung-Jin Yoon, Hajeong Lee

**Affiliations:** ^1^ Department of Internal Medicine Seoul National University Hospital Seoul National University College of Medicine Seoul Republic of Korea; ^2^ Statistics and Data Center, Samsung Biomedical Research Institute Samsung Medical Center Seoul Republic of Korea; ^3^ Department of Pediatrics Seoul National University Children's Hospital Seoul National University College of Medicine Seoul Republic of Korea; ^4^ Computer Science Department Carnegie Mellon University Pittsburgh Pennsylvania; ^5^ Department of Computer Science and Engineering Seoul National University College of Engineering Seoul Republic of Korea; ^6^ Kidney Research Institute, Seoul National University College of Medicine Seoul Republic of Korea; ^7^ Department of Biomedical Engineering Seoul National University College of Medicine Seoul Republic of Korea

**Keywords:** acute kidney injury, AKI stage, all‐cause mortality, cancer, cancer treatment

## Abstract

**Background:**

Acute kidney injury (AKI) is a critical issue in cancer patients because it is not only a morbid complication but also able to interrupt timely diagnostic evaluation or planned optimal treatment. However, the impact of AKI on overall mortality in cancer patients remains unclear.

**Methods:**

We conducted a retrospective cohort study of 67 986 cancer patients, from 2004 to 2013 to evaluate the relationship between AKI and all‐cause mortality. We used KDIGO AKI definition and grading system.

**Results:**

During 3.9 ± 3.1 years of follow‐up, 33.8% of the patients experienced AKI at least once. Among AKI events, stage 1, 2, and 3 was 71.0%, 13.8%, and 15.1%, respectively. AKI incidence was highest in hematologic malignancies, followed by urinary tract cancer, and hepatocellular carcinoma. Male sex, older age, underlying diabetes and hypertension, lower serum albumin and plasma hemoglobin, more frequent radio‐contrast exposure, entrance of clinical trials, and receiving chemotherapy were associated with AKI occurrence. AKI development was an independent risk factor for elevated mortality in cancer patients with dose‐responsive manner (Stage 1, hazard ratio [HR] 1.183, 95% confidence interval [CI] 1.145‐1.221, *P* < 0.001; Stage 2, HR 1.710, 95% CI 1.629‐1.796; Stage 3, HR 2.000, 95% CI 1.910‐2.095; No AKI, reference group) even after adjustment. This tendency was reproduced in various cancer types except thyroid cancer and in various treatment modalities, however, not shown in patients with baseline renal dysfunction.

**Conclusion:**

AKI was an independent risk factor for all‐cause mortality in overall cancer patients with dose‐responsive manner.

## 1 INTRODUCTION

Acute kidney injury (AKI) is a common, but significant complication in patients with cancer.^1^, ^2^, ^3^, ^4^ AKI is often caused by the combination of various conditions including tumor lysis syndrome (TLS), urinary tract obstruction, use of nephrotoxic drugs, and sepsis.^2^, ^5^ Although, there are not many studies on AKI in all patients with cancer, a large cohort in Denmark reported that the risk of developing AKI in 1 year was 17% for all patients with cancer.^6^ The rate of AKI and the renal replacement therapy requirements are higher in critically ill patients with cancer compared to patients without cancer.^5^, ^7^, ^8^ Patients with severe AKI, defined by a doubling of the serum creatinine, had an 8.8% and 14.6% of AKI occurrence risk at 1 and 5 years after diagnosis, respectively.^6^

Patients with poor kidney function might have a reduced likelihood of receiving optimal treatment.^1^ Recent AKI definitions such as RIFLE (Risk, Injury, Failure, Loss of kidney function, and End‐stage kidney disease) criteria^9^, ^10^ and the Acute Kidney Injury Network (AKIN) classification have emphasized that serum creatinine (sCr) differences could increase the long‐term mortality risk even in small increments.^11^, ^12^, ^13^, ^14^ Moreover, efforts to avoid renal injury progression may contribute to reducing mortality and slowing the progression to chronic kidney disease.^15^, ^16^ Thus, being aware of the development of AKI in accordance with the current AKI definitions, and the risk factors for AKI in patients with cancer would be helpful for providing proper management and adjusting treatment option.^17^

Several studies have highlighted the role of AKI on mortality in patients with hematologic malignancies receiving induction therapy,^18^ diffuse large B‐cell lymphoma,^19^ gastric cancer,^20^ bladder cancer,^21^ and lung cancer.^22^, ^23^ Additionally, the increase in mortality according to the development of AKI has not been observed in all studies due to the different patients' conditions, including overall severity, and functional status.^8^, ^24^

We aimed to evaluate AKI risk factors and the impact of AKI on mortality in patients with cancer using a large, comprehensive retrospective cohort.

## 2 METHODS

### 2.1 Study population

The Korean Ministry of Health and Welfare started a nation‐wide registry (Korea Central Cancer Registry, KCCR) for collecting data on cancer incidences and providing insurance benefits for the patients. All patients with cancer in Korea were registered in the KCCR with diagnoses based on the International Classification of Diseases 10th revision (ICD‐10).

**Figure 1:**
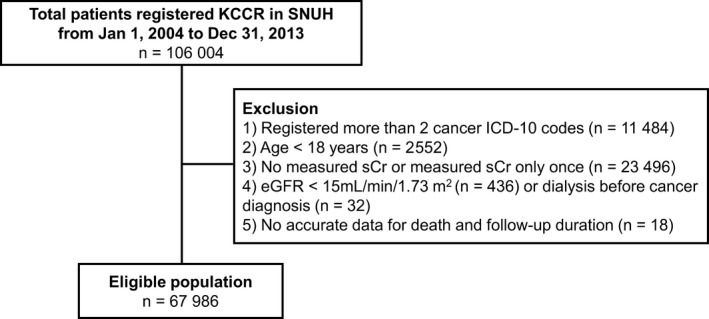
Flow diagram of the study populations. Abbreviation: KCCR, Korean central cancer registry; SNUH, Seoul National University of Hospital; ICD‐10, International Classification of Diseases 10th revision; sCr, serum creatinine; eGFR, estimated glomerular filtration rate

This study was a retrospective cohort study conducted in Seoul National University Hospital (SNUH), which is a tertiary referral hospital in Seoul, South Korea. The study population included patients who registered with KCCR at SNUH for the first time between January 1, 2004, and December 31, 2013. A total of 106 004 patients with cancer consented to study enrollment. We excluded those who (1) registered more than two types of cancers, to eliminate patients with double primary cancer, (2) were <18 years, (3) had measured sCr levels only once or did not measure sCr after the KCCR registration date, (4) had an estimated glomerular filtration rate (eGFR) <15 mL/min/1.73 m^2^ or receiving maintenance dialysis, and (5) no accurate data on death outcome. After exclusion, 67 986 patients were finally selected for the analysis (Figure 1).

### 2.2 Data collection

All data in the study were reviewed via electronic medical records (EMR) and based on the first measured values within 2 months before or after the KCCR registration date. Cancers are classified according to the Korean version of the ICD‐10. Each code is described in Table S1. The following demographic data were obtained: age, sex, and baseline body mass index (BMI). Hypertension (HTN) and diabetes mellitus (DM) were determined by formal physician diagnosis or by the documentation of patients' taking antihypertensive drugs and insulin/oral hypoglycemic agents, respectively. The number of CT scans using contrast media was investigated during the follow‐up period.

Laboratory data were collected, including hemoglobin (Hb), serum calcium, phosphorus, glucose, uric acid, protein, albumin, bilirubin, total cholesterol, triglyceride, high‐density lipoprotein (HDL), low‐density lipoprotein (LDL), blood urea nitrogen (BUN), and sCr. In terms of sCr, we extracted any and all measured sCr values requested by outpatient clinics, emergency rooms and admission department. We calculated eGFR using the Chronic Kidney Disease Epidemiology Collaboration (CKD‐EPI) equation.

We gathered information about the use and type of chemotherapeutic agent, cancer‐related surgery, and participation in new drug clinical trials. We divided the total enrolled patients into four groups: surgery and chemotherapy, surgery only, chemotherapy only, and neither surgery nor chemotherapy.

### 2.3 AKI definition

To define AKI, we set the initial sCr as the first measured sCr within 2 months before or after the KCCR registration date. If there was no sCr within that period, we defined the initial sCr as the earliest measured sCr after the date of KCCR registration. When both sCr values were present, the lower value was considered as initial sCr. Because sCr is always changeable value, thus, to define AKI events more precisely, baseline sCr was defined as the minimum value of sCr from the current time to the previous 3 weeks by shifting the reference point every 3 weeks based on the KCCR registration date.

We defined AKI events in accordance with KDIGO AKI guideline^25^ as follows: (1) an absolute increase in sCr 0.3 mg/dL from the initial or baseline sCr, (2) a percentage increase in sCr of ≥50% from the initial or baseline sCr. After developing the AKI definition, we further designated the AKI stage based on the KDIGO AKI guidelines using the ratio of sCr at the time of AKI diagnosis and initial or baseline sCr. We defined stage of AKI by meeting the following criteria based on the KDIGO AKI guideline: (1) sCr at AKI diagnosis is more than 4.0 mg/dL, stage of AKI defined as stage 3, (2) if the ratio of sCr at the time of AKI diagnosis and initial or baseline sCr was used for defining the stage of AKI, the ratio >3.0, stage 3; ratio >2.0, stage 2; ratio >1.5, stage 1, and (3) the difference between sCr at AKI diagnosis and initial or baseline sCr is more than 0.3, stage of AKI defined as stage 1.

### 2.4 Clinical outcomes

The outcome was all‐cause mortality. We collected mortality data from both an EMR review and from Statistics Korea using a unique identifier after approval from the Ministry of the Interior and Safety of Korea. The mortality data were obtained through July 2016.

### 2.5 Statistical analysis

Continuous variables are presented as mean ± standard deviation and categorical variables as actual frequencies and percentages with the Pearson's chi‐squared test. We used one‐way analysis of variance (ANOVA) or the Kruskal‐Wallis test to compare continuous variables as appropriate.

The expectation‐maximization algorithm^26^ with a bootstrap approach^27^ that produces multiple imputed datasets with plausible values, was utilized to impute missing values. The variables that were directly related to the outcome were excluded from the imputation, and this algorithm was implemented using the R package *Amelia*. The number of imputed datasets in the analysis was *m* = 5 and the combined result across five datasets were obtained using “Rubin's Rules”.^28^

We conducted a Cox regression analysis to reveal the relationship between AKI and all‐cause mortality and to calculate the hazard ratio with 95% confidence intervals (CIs). In the adjusted analysis, we introduced various potential confounders including demographics, laboratory findings, number of CT scans during follow‐up, and the occurrence of AKI. All *p*‐values were two‐sided, and values <0.05 indicated statistical significance. All statistical analyses were carried out using the R package, version 3.4.0.

## 3 RESULTS

### 3.1 Baseline characteristics of overall study participants

A total of 67 986 patients were followed for 3.9 ± 3.1 years in this study. The baseline characteristics of the enrolled patients are shown in Table 1. The mean age was 56.9 ± 13.0 years, and 50.6% of the patients were men. Individuals with DM and HTN comprised 20.3% and 9.3% of the study population, respectively. The most common cancer type was breast cancer followed by HCC and lower gastrointestinal cancers. The proportion of patients with eGFR <60 mL/min/1.73 m^2^ was approximately 10%. More than 80% of the patients were exposed to CT contrast at least once. In terms of cancer treatments, 33.7% of the patients received surgery only, 20.3% chemotherapy only, 24.3% both surgery and chemotherapy, and 21.7% received other treatments such as radiotherapy or local treatment. The patients who participated in new drug clinical trials were 1,258 (1.9%).

**Table 1:** Baseline characteristics of overall patients with cancer according to AKI stage

	Total patients	No AKI	AKI Stage 1	AKI Stage 2	AKI Stage 3	*P*
	N = 67 986	N = 44 996	N = 16 331	N = 3180	N = 3479	
Male (N, %)	34,414 (50.6)	19,585(43.5)	10,557(64.6)	1,897(59.7)	2,375(68.3)	<0.0001
Age at cancer diagnosis (years)	56.9 ± 13.0	56.9 ± 13.0	59.7 ± 12.8	59.9 ± 12.9	59.3 ± 12.3	<0.0001
Comorbidities (N, %)						
Hypertension	13,789 (20.3)	7,602 (16.9)	4,355 (26.7)	880 (27.7)	952 (27.4)	<0.0001
Diabetes	6,312 (9.3)	3,143 (7.0)	2,155 (13.2)	458 (14.4)	556 (16)	<0.0001
Smoking (N, %)						
Current smoker	8,474 (12.5)	4,705 (10.5)	2,697 (16.5)	492(15.5)	580 (16.7)	<0.0001
BMI (kg/m^2^)	23.6 ± 3.2	23.6 ± 3.2	23.5 ± 3.3	23.3 ± 3.4	23.6 ± 3.3	0.002
SBP (mmHg)	123.5 ± 16.4	123.1 ± 16.1	124.2 ± 16.7	122.9 ± 16.8	125.2 ± 17.8	<0.0001
DBP (mmHg)	76.5 ± 11.1	76.6 ± 11.1	76.6 ± 11.2	76.0 ± 11.2	76.7 ± 11.2	0.690
Cancer (N, %)						
Thyroid cancer	6,146 (9.0)	5,677 (12.6)	390 (2.4)	47 (1.5)	32 (0.9)	<0.0001
Breast cancer	10,820 (15.9)	9,513 (21.1)	1,079 (6.6)	125 (3.9)	103 (3)	
Head and neck cancer	1,253 (1.8)	821 (1.8)	356 (2.2)	45 (1.4)	31 (0.9)	
Central nervous system	1,365 (2.0)	909 (2.0)	388 (2.4)	44 (1.4)	24 (0.7)	
Stomach	9,547 (14)	6,733 (15.0)	2,179 (13.3)	347 (10.9)	288 (8.3)	
Colorectal and anal cancer	7,385 (10.9)	4,772 (10.6)	1,979 (12.1)	298 (9.4)	336 (9.7)	
Pancreatic cancer	1,638 (2.4)	878 (2.0)	522 (3.2)	141 (4.4)	97 (2.8)	
CBD and GB cancer	1,287 (1.9)	670 (1.5)	432 (2.6)	88 (2.8)	97 (2.8)	
Hepatocellular carcinoma	7,980 (11.7)	3,688 (8.2)	2,364 (14.5)	803 (25.3)	1,125 (32.3)	
Respiratory tract cancer	5,855 (8.6)	3,239 (7.2)	2,140 (13.1)	284 (8.9)	192 (5.5)	
Kidney and urinary tract cancer	3,640 (5.4)	1,667 (3.7)	1,505 (9.2)	170 (5.3)	298 (8.6)	
Female genital organ cancer	2,341 (3.4)	1,503 (3.3)	561 (3.4)	144 (4.5)	133 (3.8)	
Male genital organ cancer	2,493 (3.7)	1,685 (3.7)	591 (3.6)	48 (1.5)	169 (4.9)	
Hematologic malignancy	3,226 (4.7)	1,440 (3.2)	923 (5.7)	432 (13.6)	431 (12.4)	
Bone, joint, and soft tissue cancer	978 (1.4)	618 (1.4)	289 (1.8)	36 (1.1)	35 (1.0)	
Skin cancer and melanoma	696 (1.0)	506 (1.1)	158 (1.0)	22 (0.7)	10 (0.3)	
Other cancer	1,336 (2.0)	677 (1.5)	475 (2.9)	106 (3.3)	78 (2.2)	
Laboratory data						
eGFR at diagnosis (mL/min/1.73 m^2^)	82.3 ± 17.8	83.2 ± 16.1	80.6 ± 19.7	82.0 ± 20.3	77.5 ± 24.4	<0.0001
≥90	22,973 (33.8)	15,230 (33.8)	5,441 (33.3)	1,157 (36.4)	1,145 (32.9)	
60‐89	38,239 (56.2)	26,588 (59.1)	8,476 (51.9)	1,577 (49.6)	1,598 (45.9)	
45‐59	5,068 (7.5)	2,706 (6.0)	1,681 (10.3)	329 (10.3)	352 (10.1)	
30‐45	1,245 (1.8)	402 (0.9)	559 (3.4)	96 (3.0)	188 (5.4)	
15‐30	461 (0.7)	70 (0.2)	174 (1.1)	21 (0.7)	196 (5.6)	
Creatinine (mg/dL)	0.9 ± 0.3	0.9 ± 0.2	1.0 ± 0.3	0.9 ± 0.3	1.1 ± 0.5	<0.0001
Albumin (g/dL)	4.1 (0.5)	4.2 ± 0.4	3.9 ± 0.6	3.7 ± 0.7	3.7 ± 0.7	<0.0001
Bilirubin (mg/dL)	1.0 ± 1.8	0.9 ± 1.2	1.2 ± 2.2	1.4 ± 2.6	1.7 ± 3.5	<0.0001
Uric acid (mg/dL)	4.8 ± 1.5	4.7 ± 1.4	5.0 ± 1.7	4.8 ± 1.8	5.1 ± 1.9	<0.0001
WBC (×10^3^/µL)	7.1 ± 8.2	6.8 ± 6.8	7.6 ± 10.0	8.1 ± 13.1	7.4 ± 7.0	<0.0001
Hemoglobin (g/dL)	13.1 ± 2.0	13.3 ± 1.8	12.9 ± 2.1	12.2 ± 2.3	12.3 ± 2.4	<0.0001
Platelet (×10^3^/µL)	236.6 ± 92.9	240.4 ± 83.1	235.7 ± 101.3	221.8 ± 130.2	208.6 ± 113.7	<0.0001
Total cholesterol (mg/dL)	176.8 ± 41.6	180.9 ± 38.3	171.2 ± 44.3	164.3 ± 50.2	164.8 ± 49.3	<0.0001
CT (N, %)						
No CT	11,689 (17.2)	9,606 (21.3)	1,544 (9.5)	205 (6.4)	334 (9.6)	<0.0001
>0~1/year	15,965 (23.5)	12,370 (27.5)	2,790 (17.1)	370 (11.6)	435 (12.5)	
>1~3/year	19,683 (29.0)	13,219 (29.4)	5,081 (31.1)	729 (22.9)	654 (18.8)	
≥3/year	20,649 (30.4)	9,801 (21.8)	6,916 (42.3)	1,876 (59.0)	2,056 (59.1)	
Clinical trial (N, %)	1,258 (1.9)	1,258 (1.9)	474 (2.9)	111 (3.5)	97 (2.8)	<0.0001
Cancer treatment (N, %)						
Surgery only	22,941 (33.7)	17,246 (38.3)	4,596 (28.1)	509 (16)	590 (17.0)	<0.0001
Surgery and chemotherapy	16,496 (24.3)	10,074 (22.4)	4,692 (28.7)	897 (28.2)	833 (23.9)	
Chemotherapy only	13,816 (20.3)	6,276 (13.9)	4,717 (28.9)	1,320 (41.5)	1,503 (43.2)	
No surgery and chemotherapy	14,733 (21.7)	11,400 (25.3)	2,326 (14.2)	454 (14.3)	553 (15.9)	

Abbreviations: AKI, acute kidney injury; BMI, body mass index; SBP, systolic blood pressure; DBP, diastolic blood pressure; CBD, common bile duct; GB, gallbladder; eGFR, estimated glomerular filtration rate; WBC, white blood cell; CT, computed tomography

### 3.2 Comparisons of patients' characteristics according to AKI stage

Baseline characteristics according to the AKI stages was described in Table 1. The proportions of patients with No AKI, AKI stage 1, 2, and 3 were 66.2%, 24.0%, 4.7%, and 5.1%, respectively, and the median time to the first AKI event was 106 days (interquartile range 25‐476 days). The more severe the AKI, the lower the levels of the initial eGFR, serum albumin, and platelet counts. The AKI stage was positively correlated with radio‐contrast exposure counts. Additionally, patients with AKI received more chemotherapy and were enrolled in more clinical trials than patients without AKI.

**Figure 2:**
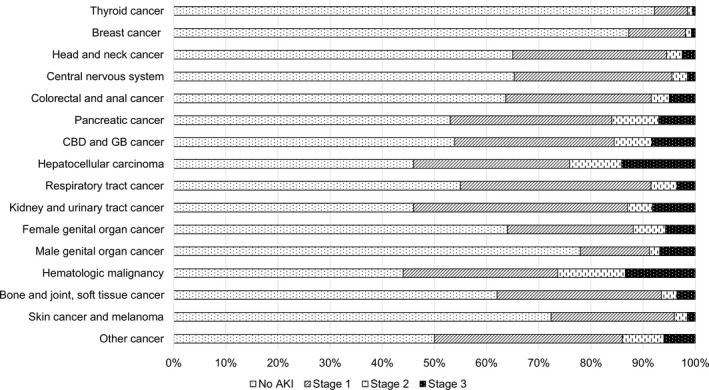
Description of AKI stage according to cancer type. Abbreviation: AKI, acute kidney injury; CBD, common bile duct; GB, gallbladder

AKI stage according to cancer type is summarized in Figure 2. Overall AKI incidence was highest with hematologic malignancies. More than half of patients with these three types of cancer experienced an AKI event after their cancer diagnosis. Severe AKI with stage 3 was shown most in HCC (14.1%), followed by hematologic malignancy (13.4%), kidney and urinary tract cancer (8.2%), male (6.8%), and female (5.68%) genital organ cancer. The number of patients who never developed AKI during the follow‐up period was larger in those with thyroid cancer (92.4%), followed by breast cancer (87.92%).

### 3.3 Risk Factors for AKI development

We performed a logistic regression analysis to find risk factors for AKI development and the results are summarized in Table S2. Male sex, older age, HTN, DM, high mean arterial pressure, and clinical trial participation were revealed to be independent risk factors for AKI occurrence in all patients with cancer. Kidney and urinary tract cancer had the highest rates of AKI, followed by hematologic. Lower serum albumin and hemoglobin, higher serum bilirubin and uric acid, multiple CT exposures, and chemotherapy also increased the occurrence of AKI.

Lower eGFR was also an important risk factor for AKI in patients with cancer, especially if the eGFR was less than 60 mL/min/1.73 m^2^. These patients showed a step‐wise increase in AKI risk with a decrease in baseline renal function (eGFR 45‐59 mL/min/1.73 m^2^, adjusted odds ratio [aOR] 1.302, 95% CI 2.332‐3.129, *P *< 0.01; 30‐45 mL/min/1.73 m^2^, aOR 2.701, 95% CI 2.332‐3.129, *P *< 0.01; 15‐30 mL/min/1.73 m^2^, aOR 7.583, 95% CI 5.661‐10.157, ≥90 mL/min/1.73 m^2^, reference group). Interestingly, however, this tendency was not observed in patients with eGFR 60‐89 mL/min/1.73 m^2^, who showed a rather reduced risk of AKI compared with the reference eGFR group (eGFR 60‐89 mL/min/1.73 m^2^, aOR 0.809, 95% CI 0.774‐0.845, *P *< 0.01).

### 3.4 AKI occurrence and all‐cause mortality

During the observation period, a total of 23 022 (33.9%) deaths occurred. AKI occurrence was associated with increased mortality in a step‐wise manner in the univariate analysis. When adjusted for age, sex, and cancer type (Model 1), this dose‐responsive relationship remained significant. We additively adjusted for the co‐morbidities and various laboratory results (Model 2), as well as the sequentially contrast‐enhanced CT counts, inclusion of clinical trials, and cancer treatments (Model 3). Consequentially, AKI stage was an independent risk factor for elevated mortality in patients with cancer in a dose‐responsive manner (Table 2).

**Table 2:** Multivariable Cox proportional hazard analysis for mortality in overall participants

	Univariate analysis		Model 1^a^		Model 2^b^		Model 3^c^	
AKI stage	HR (95% CI)	*P*	HR (95% CI)	*P*	HR (95% CI)	*P*	HR (95% CI)	***P***
No AKI	Reference		Reference		Reference		Reference	
Stage 1	2.641 (2.540‐2.746)	<0.001	1.752 (1.700‐1.806)	<0.001	1.495 (1.449‐1.543)	<0.001	1.183 (1.145‐1.221)	<0.001
Stage 2	4.788 (4.605‐4.978)	<0.001	3.037 (2.900‐3.180)	<0.001	2.397 (2.283‐2.515)	<0.001	1.710 (1.629‐1.796)	<0.001
Stage 3	5.322 (5.119‐5.533)	<0.001	3.654 (3.498,3.817)	<0.001	2.889 (2.758‐3.025)	<0.001	2.000 (1.910‐2.095)	<0.001

Abbreviations: HR, hazard ratio; CI, confidence interval; eGFR, estimated glomerular filtration rate; CT. computed tomography; AKI, acute kidney injury.

a AKI stage adjusted for age, age, cancer type.

b AKI stage adjusted for Model 1 variables + hypertension, diabetes, smoking, body mass index, mean arterial pressure, eGFR, albumin, bilirubin, uric acid, hemoglobin, and total cholesterol.

c AKI stage adjusted for Model 2 variables + CT count, clinical trial, and cancer treatment.

**Figure 3:**
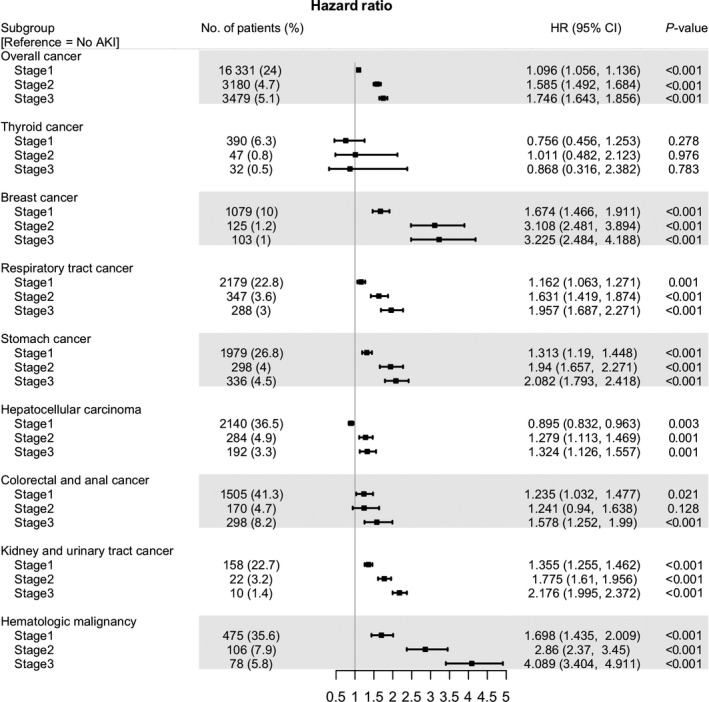
Forest plot of multivariable COX analysis according to cancer type. Abbreviation: AKI, acute kidney injury; HR, hazard ratio; CI, confidential interval

Figure 3 summarizes the results of a subgroup analysis according to the various cancer types. AKI stage was an important risk factor in most cancer types, except thyroid cancer. Moreover, AKI severity had a dose‐responsive association with all‐cause mortality, and this tendency was most prominent in the hematologic malignancies, and kidney and urinary tract cancer.

**Figure 4:**
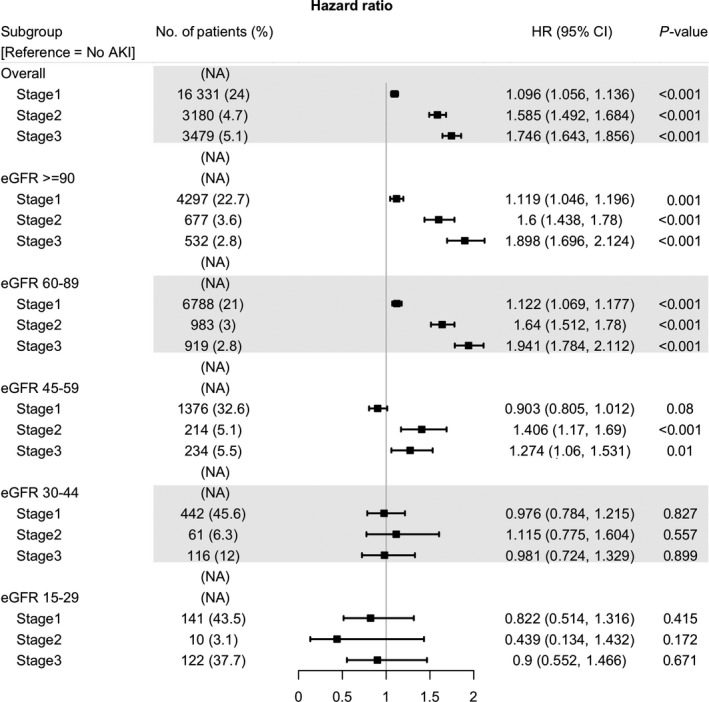
Forest plot of multivariable COX analysis according to eGFR at cancer diagnosis. Abbreviation: eGFR, estimated glomerular filtration rate; HR, hazard ratio; CI, confidential interval

Figure 4 shows that AKI affected all‐cause mortality in a dose‐responsive manner in patients with cancer with preserved baseline renal function (eGFR ≥ 60 mL/min/1.73 m^2^). However, it did not show an effect in those with advanced baseline renal impairments (eGFR 15‐45 mL/min/1.73 m^2^). Patients with moderate renal dysfunction (eGFR 45‐60 mL/min/1.73 m^2^) had elevated mortality risk only when they had AKI higher than stage 2.

**Figure 5:**
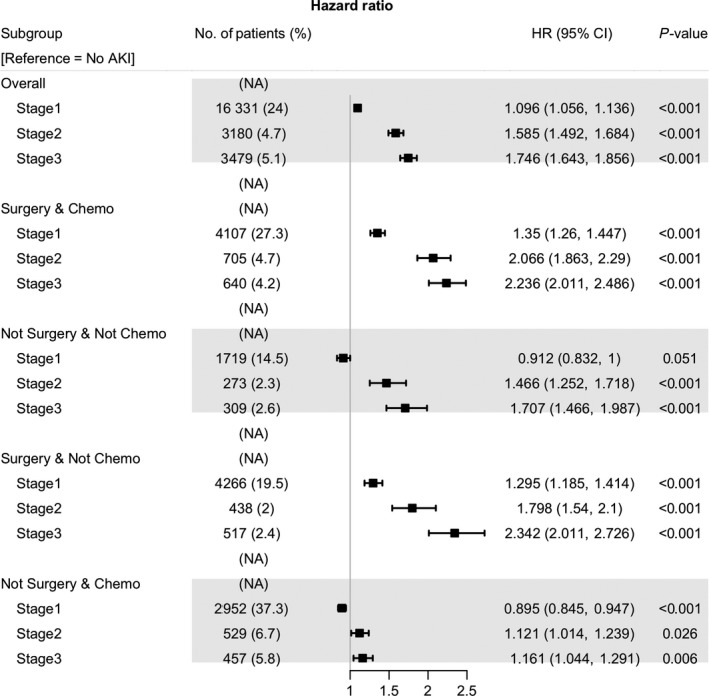
Forest plot of multivariable COX analysis according to cancer treatment. Abbreviation: AKI, acute kidney injury; HR, hazard ratio; CI, confidential interval

Finally, we analyzed the influence of AKI on mortality according to the type of cancer treatment (Figure 5). A dose‐response association between all‐cause mortality and AKI stage were observed in nearly all the subgroups. In the chemotherapy‐only group, all‐cause mortality was lower in patients in AKI stage 1 than in patients without AKI (AKI stage 1 in the chemotherapy‐only group, HR 0.895, 95% CI 0.845‐0.947, *P *< 0.01). The difference in the HRs was different for each subgroup, and the mortality risk according to AKI stage was higher in patients undergoing surgery.

## 4 DISCUSSION

In this large‐scale study, we found that AKI severity determined by the KDIGO AKI staging method, was associated with increased mortality in a dose‐responsive manner in all patients with cancer and all cancer types except for thyroid cancer. Interestingly, the impact of AKI severity on mortality was evident only in the group with eGFR ≥ 60 mL/min/1.73 m^2^. Patients with cancer and moderate renal dysfunction (eGFR 45‐60 mL/min/1.73 m^2^) had an increased mortality risk only if they had stage 2 or 3 AKI.

Compared to the general population, with an AKI incidence of 1811 per million population,^29^ there is no exact incidence data for all patients with cancer. We assumed that these patients would have a high incidence of AKI. Previous studies have shown that the incidence of AKI was higher in patients with cancer than in patients without cancer.^29^ In our study, about one‐third of the patients with cancer experienced an AKI event at least once. As reported in previous studies, hematologic malignancy was the most common type of cancer associated with AKI development (55.4%), which was a similar incidence in our study.^30^, ^31^

Besides well‐known AKI events, including TLS, chemotherapy, and contrast‐induced nephropathy (CIN) might be associated with higher incidence of AKI in cancer patients. However, the incidence can be controlled as physicians can modulate the number of contrast CT scans or adjust chemotherapeutic agent dosages in consideration of the individual patients' AKI risk. Many physicians have provided prevention strategies for CIN including volume repletion with or without use of N‐acetylcysteine, although the effects are still controversial.^32^ Reducing contrast exposures might alleviate AKI risk in patients with cancer. Chemotherapy, an essential therapeutic agent, is an important risk factor for AKI.^24^ Traditional chemotherapeutic agents cause AKI via thrombotic microangiopathy, crystalline nephropathy, acute tubular injury, and tubulointerstitial injury.^24^ Additionally, emerging targeting agents should be evaluated according to their kidney toxicities,^33^ in spite of their potentially life‐saving capacity. Meticulous dose adjustment based on patients' renal function and AKI risk factors is warranted in the prescription of chemotherapeutic agents.

One of the interesting findings was that the risk of AKI was the lowest in patients with eGFRs of 60‐89 mL/min/1.73 m^2^, rather than eGFRs ≥ 90 mL/min/1.73 m^2^. Conventional eGFR was calculated based on age, sex, and serum creatinine. Considering age and cachexia in patients with cancer, eGFR ≥ 90 mL/min/1.73 m^2^ is likely to be an overestimation of their actual renal function because of the decreased muscle mass in patients with cancer. Recently, cystatin C not influenced by protein catabolism, dietetic factors, or muscle mass is emerging biomarkers for renal function. Previously, the use of cystatin C was limited for fear that its concentration might be influenced by the extent of the cancer,^8^ but recent studies revealed that there is no relationship between cystatin C and tumor burden.^34^ Further studies are needed to assess the usefulness of cystatin C in patients with cancer.

Although, there have been many reports that AKI is associated with all‐cause mortality in hospitalized patients,^35^, ^36^, ^37^ there is a lack of research on whether this association applies in patients with cancer. In previous studies of patients with cancer who were treated in the intensive care unit (ICU), mortality was increased according to the RIFLE risk category in a dose‐responsive manner.^22^ This relationship was observed in nearly all cancer types except thyroid cancer, presumably due to its female predominance, the lower proportion of patients receiving chemotherapy, follow‐up using ultrasonography rather than contrast‐enhanced CT, and the relatively lower risk of surgery.

In a multivariable Cox analysis, dose‐responsiveness was observed between all‐cause mortality and AKI severity, except in the chemotherapy only group (Figure 5), which, in our cohort comprised patients with HCC and respiratory tract cancer (data not shown). Transarterial chemoembolization (TACE) has been used as the most common therapeutic method in real‐life clinical practice in South Korea.^38^ Patients who have received long‐term repeated TACE eventually develop lung or other organ metastasis, and they are switched to systemic chemotherapy. Therefore, the survival rate of patients treated with chemotherapy‐only might be higher than those of other groups. One of the hypotheses regarding this phenomenon is due to TLS. Hematologic malignancy and small cell lung cancer are common cancers that exhibit TLS because they have good response to chemotherapy‐only.^39^ A large number of patients who are diagnosed with hematologic malignancy received chemotherapy‐only (60%) in our cohort.

Recently, many patients with cancer are increasingly being treated as outpatients in chemotherapy day units rather than hospitalization. Therefore, the clinical course in the outpatient setting should also be included for an adequate assessment of AKI in patients with cancer. However, most of the studies regarding AKI were conducted in patients who were hospitalized, including in the ICU, and the mortality of critically ill patients with cancer reached 14%‐30%.^2^ We confirmed that the risk of all‐cause mortality increased as the severity of AKI increased based on sCr in all clinical settings mentioned above.

This study has several limitations. First, although staging is one of the important factors in the survival of patients with cancer, we could not take this into account in our study. Therefore, we divided them into locoregional versus systemic treatment according to chemotherapy and surgery. The surgery‐only group was likely to have received curative surgery, the chemotherapy‐only group was likely to receive palliative chemotherapy except those with hematologic malignancies. Additionally, the patients who had surgery and chemotherapy performed simultaneously were likely to have received adjuvant or neo‐adjuvant chemotherapy. Second, we could not fully evaluate all the factors influencing AKI events as covariates, including urinary tract obstruction, the usage of nonsteroidal anti‐inflammatory drugs, and other radiologic interventions that used contrast media, with the exception of CT. Third, when we evaluated AKI development, we only measured sCr once. Therefore, the sCr may not indicate true baseline renal function of each patient, which means that when sCr was higher than normal level, it was difficult to know whether the sCr meant AKI on chronic kidney disease (CKD) or CKD only. In addition, because each patient had a different interval for measuring the sCr during the follow‐up period, patients who frequently measure sCr were more likely to develop AKI. Last, we dealt with only the initial AKI events instead of recurrent or continuous events.

We found that >33% of the patients with cancer experienced an AKI event. Moreover, we demonstrated that AKI is an important risk factor for all‐cause mortality in all patients with cancer in a dose‐responsive manner. Physicians and researchers should not only focus on therapeutic options for cancer, but also optimize maintenance of kidney health during a period of continuously improving cancer survival.

## 5 ETHICS STATEMENT

The study protocol was approved by the institutional review board at SNUH (H‐1509‐051‐702). The study protocol was conducted in accordance with the principles of the Declaration of Helsinki.

## CONFLICT OF INTEREST

None declared.

## AUTHORS' CONTRIBUTIONS

All authors were involved in drafting the article or revising it critically for important intellectual content, and all authors approved the final version to be submitted for publication. Study design: Hajeong Lee, Hee Gyung Kang , and Hyung‐Jin Yoon; Acquisition of Data: Hajeong Lee, Eunjeong Kang, Younglee Jung, Namyong Park, Peong Gang Park, Dong Ki Kim, Kook‐Hwan Oh, Kwon Wook Joo, and Yon Su Kim; Data analysis: Minsu Park and Namyong Park; Writing the manuscript: Eunjeong Kang; Review, revision, and final approval: Hajeong Lee. The results presented in this paper have not been published previously in their entirety or in part, except in abstract form.

## References

[1] Lameire NH , Flombaum CD , Moreau D , Ronco C . Acute renal failure in cancer patients. Ann Med. 2005;37:13‐25. 15902843 10.1080/07853890510007205

[2] Campbell GA , Hu D , Okusa MD . Acute kidney injury in the cancer patient. Adv Chronic Kidney Dis. 2014;21:64‐71. 24359988 10.1053/j.ackd.2013.08.002

[3] Humphreys BD , Soiffer RJ , Magee CC . Renal failure associated with cancer and its treatment: an update. J Am Soc Nephrol. 2005;16:151‐161. 15574506 10.1681/ASN.2004100843

[4] Cohen EP . Cancer and the kidney: the frontier of nephrology and oncology. Oxford: Oxford University Press; 2010.

[5] Darmon M , Ciroldi M , Thiery G , Schlemmer B , Azoulay E . Clinical review: Specific aspects of acute renal failure in cancer patients. Critical Care. 2006;10:211‐211. 16677413 10.1186/cc4907 PMC1550893

[6] Christiansen CF , Johansen MB , Langeberg WJ , Fryzek JP , Sorensen HT . Incidence of acute kidney injury in cancer patients: a Danish population‐based cohort study. Eur J Intern Med. 2011;22:399‐406. 21767759 10.1016/j.ejim.2011.05.005

[7] Benoit DD , Hoste EA , Depuydt PO , et al. Outcome in critically ill medical patients treated with renal replacement therapy for acute renal failure: comparison between patients with and those without haematological malignancies. Nephrol Dial Transplant. 2005;20:552‐558. 15671075 10.1093/ndt/gfh637

[8] Darmon M , Thiery G , Ciroldi M , et al. Intensive care in patients with newly diagnosed malignancies and a need for cancer chemotherapy. Crit Care Med. 2005;33:2488‐2493. 16276171 10.1097/01.ccm.0000181728.13354.0a

[9] Ostermann M , Chang RW . Acute kidney injury in the intensive care unit according to RIFLE. Crit Care Med. 2007;35:1837–1843; quiz 1852. 17581483 10.1097/01.CCM.0000277041.13090.0A

[10] Bagshaw SM , George C , Dinu I , Bellomo R . A multi‐centre evaluation of the RIFLE criteria for early acute kidney injury in critically ill patients. Nephrol Dial Transplant. 2008;23:1203–1210. 17962378 10.1093/ndt/gfm744

[11] Joannidis M , Metnitz B , Bauer P , et al. Acute kidney injury in critically ill patients classified by AKIN versus RIFLE using the SAPS 3 database. Intensive Care Med. 2009;35:1692–1702. 19547955 10.1007/s00134-009-1530-4

[12] Wilson FP , Bansal AD , Jasti SK , et al. The impact of documentation of severe acute kidney injury on mortality. Clin Nephrol. 2013;80:417–425. 24075024 10.5414/CN108072 PMC4018223

[13] Thakar CV , Christianson A , Freyberg R , Almenoff P , Render ML . Incidence and outcomes of acute kidney injury in intensive care units: a Veterans Administration study. Crit Care Med. 2009;37:2552–2558. 19602973 10.1097/CCM.0b013e3181a5906f

[14] Murugan R , Kellum JA . Acute kidney injury: what's the prognosis? Nat Rev Nephrol. 2011;7:209–217. 21343898 10.1038/nrneph.2011.13 PMC3547642

[15] Chawla LS , Amdur RL , Amodeo S , Kimmel PL , Palant CE . The severity of acute kidney injury predicts progression to chronic kidney disease. Kidney Int. 2011;79:1361–1369. 21430640 10.1038/ki.2011.42 PMC3257034

[16] Sawhney S , Mitchell M , Marks A , Fluck N , Black C . Long‐term prognosis after acute kidney injury (AKI): what is the role of baseline kidney function and recovery? A systematic review. BMJ Open. 2015;5:e006497. 10.1136/bmjopen-2014-006497 PMC4289733 25564144

[17] Lameire N , Vanholder R , Van Biesen W , Benoit D . Acute kidney injury in critically ill cancer patients: an update. Crit Care. 2016;20:209. 27480256 10.1186/s13054-016-1382-6 PMC4969681

[18] Lahoti A , Kantarjian H , Salahudeen AK , et al. Predictors and outcome of acute kidney injury in patients with acute myelogenous leukemia or high‐risk myelodysplastic syndrome. Cancer. 2010;116:4063–4068. 20564156 10.1002/cncr.25306 PMC4181579

[19] Hong J , Lee S , Chun G , et al. Baseline renal function as a prognostic indicator in patients with newly diagnosed diffuse large B‐cell lymphoma. Blood Res. 2016;51:113–121. 27382556 10.5045/br.2016.51.2.113 PMC4931929

[20] Kim CS , Bae EH , Ma SK , Kweon SS , Kim SW . Impact of transient and persistent acute kidney injury on chronic kidney disease progression and mortality after gastric surgery for gastric cancer. PLoS One. 2016;11:e0168119. 27936153 10.1371/journal.pone.0168119 PMC5148089

[21] Kwon T , Jeong IG , Lee C , et al. Acute kidney injury after radical cystectomy for bladder cancer is associated with chronic kidney disease and mortality. Ann Surg Oncol. 2016;23:686–693. 26442922 10.1245/s10434-015-4886-4

[22] Libório AB , Abreu K , Silva, Jr. GB , et al. Predicting hospital mortality in critically ill cancer patients according to acute kidney injury severity. Oncology. 2011;80:160–166. 21677465 10.1159/000329042

[23] Licker M , Cartier V , Robert J , et al. Risk factors of acute kidney injury according to RIFLE criteria after lung cancer surgery. Ann Thorac Surg. 2011;91:844–850. 21353011 10.1016/j.athoracsur.2010.10.037

[24] Rosner MH , Perazella MA . Acute kidney injury in patients with cancer. N Engl J Med. 2017;376:1770–1781. 28467867 10.1056/NEJMra1613984

[25] Khwaja A . KDIGO clinical practice guidelines for acute kidney injury. Nephron Clin Pract. 2012;120:c179–c184. 22890468 10.1159/000339789

[26] Dempster AP , Laird NM , Rubin DB . Maximum likelihood from incomplete data via the EM algorithm. J R Stat Soc Ser B Stat Methodol. 1977;39:1–38.

[27] Honaker J , King G . What to Do about missing values in time‐series cross‐section data. Am J Polit Sci. 2010;54:561–581.

[28] Multiple D . Imputation for Non‐response in Surverys. New York: Wiley and Sons; 1987.

[29] Salahudeen AK , Doshi SM , Pawar T , Nowshad G , Lahoti A , Shah P . Incidence rate, clinical correlates, and outcomes of AKI in patients admitted to a comprehensive cancer center. Clin J Am Soc Nephrol. 2013;8:347–354. 23243268 10.2215/CJN.03530412 PMC3586962

[30] Khalil MA , Latif H . Acute kidney injury in lymphoma: a single centre experience. Int J Nephrol. 2014;2014:272961. 24639896 10.1155/2014/272961 PMC3930139

[31] Darmon M , Vincent F , Canet E , et al. Acute kidney injury in critically ill patients with haematological malignancies: results of a multicentre cohort study from the Groupe de Recherche en Reanimation Respiratoire en Onco‐Hematologie. Nephrol Dial Transplant. 2015;30:2006–2013. 26597921 10.1093/ndt/gfv372 PMC4832999

[32] Davenport MS , Cohan RH , Ellis JH . Contrast media controversies in 2015: imaging patients with renal impairment or risk of contrast reaction. Am J Roentgenol. 2015;204:1174–1181. 25730301 10.2214/AJR.14.14259

[33] Perazella MA . Checkmate: kidney injury associated with targeted cancer immunotherapy. Kidney Int. 2016;90:474–476. 27521108 10.1016/j.kint.2016.05.024

[34] Okura T , Jotoku M , Irita J , et al. Association between cystatin C and inflammation in patients with essential hypertension. Clin Exp Nephrol. 2010;14:584–588. 20809110 10.1007/s10157-010-0334-8

[35] Wang HE , Muntner P , Chertow GM , Warnock DG . Acute kidney injury and mortality in hospitalized patients. Am J Nephrol. 2012;35:349–355. 22473149 10.1159/000337487 PMC3362180

[36] Chertow GM , Burdick E , Honour M , Bonventre JV , Bates DW . Acute kidney injury, mortality, length of stay, and costs in hospitalized patients. J Am Soc Nephrol. 2005;16:3365–3370. 16177006 10.1681/ASN.2004090740

[37] Coca SG , Yusuf B , Shlipak MG , Garg AX , Parikh CR . Long‐term risk of mortality and other adverse outcomes after acute kidney injury: a systematic review and meta‐analysis. Am J Kidney Dis. 2009;53:961–973. 19346042 10.1053/j.ajkd.2008.11.034 PMC2726041

[38] Yoon SK , Chun HG . Status of hepatocellular carcinoma in South Korea. Chin Clin Oncol. 2013;2:39. 25841918 10.3978/j.issn.2304-3865.2013.11.08

[39] Howard SC , Jones DP , Pui C‐H . The tumor lysis syndrome. N Engl J Med. 2011;364:1844–1854. 21561350 10.1056/NEJMra0904569 PMC3437249

