# Supplementary material for: Acute kidney injury predicts all‐cause mortality in patients with cancer
Source: Cancer Med. 2019 Apr 9;8(6):2740–50. doi: 10.1002/cam4.2140 (PMC6558474; doi:10.1002/cam4.2140)
Supplement: Supplementary file 1 [file CAM4-8-2740-s001.docx]

**Supplementary**

**Table S1. Classification of the ICD-10 codes**

| **Cancer classification** | **Sub-classification** | **ICD-10 codes** |
| --- | --- | --- |
| **Head and neck cancer** | Tongue | C01-C02 |
|  | Pharynx cancer | C10, C11, C13 |
|  | Larynx cancer | C32 |
|  | Eye cancer | C69 |
|  | Parotid, salivary gland | C07, C08 |
|  | Tonsil | C09 |
|  | Other head and neck cancers | C00, C03, C04, C05, C06, C12, C14, C30, C31 |
| **Central nervous system** | Meninges | C70 |
|  | Brain | C71 |
|  | Spinal cord | C72 |
| **Gastrointestinal tract** | Esophagus | C15 |
|  | Stomach | C16 |
|  | Colorectal cancer | C18, C19, C20 |
|  | Anal cancer | C21 |
|  | Liver | C22 |
|  | Pancreas | C25 |
|  | Biliary tract (common bile duct) | C24 |
|  | Gallbladder cancer | C23 |
|  | Other gastrointestinal cancer | C17, C26 |
| **Respiratory tract cancer** | Lung | C34 |
|  | Other respiratory tract cancer | C33, C39 |
|  | Heart, mediastinum, pleura | C38 |
| **Thymus** |  | C37 |
| **Bones and joints** |  | C40, C41 |
| **Soft tissue** | Nervous system | C47 |
|  | Retroperitoneum | C48 |
|  | Connective tissue, soft tissue | C49 |
| **Skin cancer** |  | C44 |
| **Melanoma** |  | C43 |
| **Mesothelioma** |  | C45 |
| **Breast cancer** |  | C50 |
| **Female genital organ cancer** | Cervical cancer | C53, C54, C55 |
|  | Ovarian cancer | C56 |
|  | Placental neoplasm | C58 |
|  | Other female genital tract cancers | C51, C52, C57 |
| **Male genital organ cancer** | Prostate cancer | C61 |
|  | Testis | C62 |
|  | Other male genital tract cancers | C60, C63 |
| **Kidney and urinary tract cancer** | Kidney | C64, C65 |
|  | Bladder | C67 |
|  | Other urinary tract and kidney cancer | C66, C68 |
| **Endocrine organ** | Thyroid cancer | C73 |
|  | Adrenal gland | C74 |
|  | Other endocrine cancer | C75 |
| **MUO** |  | C80 |
| **Hematologic malignancy** | Hodgkin’s lymphoma | C81 |
|  | Non-Hodgkin’s lymphoma | C82-C88 |
|  | Multiple myeloma | C90 |
|  | Lymphoid leukemia | C91 |
|  | Myeloid and monocytic leukemia | C92 |
|  | Other leukemia | C93, C94, C95 |
|  | Other lymphoid, hematopoietic related tissue | C96 |

*Abbreviations*: ICD-10: international classification of diseases 10^th^ revision; MUO: metastasis of unknown origin

**Table S2. Multivariable logistic regression analysis models for AKI development in overall participants**

| **Variables** | | **multivariate analysis^a^** | |
| --- | --- | --- | --- |
|  |  | **Adjusted OR (95% CI)** | ***P*** |
| **Sex (reference. female)** | | 1.400 (1.329-1.476) | <.01 |
| **Age at diagnosis** | | 1.008 (1.007-1.010) | <.01 |
| **Comorbidities** | |  |  |
|  | Hypertension | 1.251 (1.192-1.314) | <.01 |
|  | Diabetes | 1.290 (1.212-1.374) | <.01 |
| **Current smoker** | | 1.042 (0.980-1.108) | .17 |
| **Body mass index** | | 1.000 (0.994-1.007) | .94 |
| **Mean arterial pressure** | | 1.003 (1.001-1.005) | <.01 |
| **Cancer type** | |  |  |
|  | Thyroid cancer | reference |  |
|  | Breast cancer | 0.929 (0.824-1.048) | .23 |
|  | Head and neck cancer | 2.440 (2.071-2.875) | <.01 |
|  | Central nervous system | 4.312 (3.683-5.047) | <.01 |
|  | Stomach | 1.834 (1.635-2.057) | <.01 |
|  | Colorectal and anal cancer | 1.520 (1.349-1.712) | <.01 |
|  | Pancreatic cancer | 2.093 (1.796-2.440) | <.01 |
|  | CBD and GB cancer | 2.397 (2.035-2.823) | <.01 |
|  | Hepatocellular carcinoma | 2.697 (2.390-3.044) | <.01 |
|  | Respiratory tract cancer | 2.222 (1.968-2.509) | <.01 |
|  | Kidney and urinary tract cancer | 6.493 (5.732-7.355) | <.01 |
|  | Female genital organ cancer | 2.515 (2.188-2.890) | <.01 |
|  | Male genital organ cancer | 3.408 (2.968-3.913) | <.01 |
|  | Hematologic malignancy | 5.085 (4.455-5.804) | <.01 |
|  | Bone, joint and soft tissue cancer | 3.346 (2.811-3.982) | <.01 |
|  | Skin cancer and melanoma | 2.500 (2.030-3.078) | <.01 |
|  | Other cancer | 3.257 (2.781-3.816) | <.01 |
| **eGFR at diagnosis** | |  |  |
|  | ≥90 mL/min/1.73m^2^ | reference |  |
|  | 60-89 mL/min/1.73m^2^ | 0.809 (0.774-0.845) | <.01 |
|  | 45-59 mL/min/1.73m^2^ | 1.302 (1.202-1.410) | <.01 |
|  | 30-44 mL/min/1.73m^2^ | 2.701 (2.332-3.129) | <.01 |
|  | 15-29 mL/min/1.73m^2^ | 7.583 (5.661-10.157) | <.01 |
| **Laboratory data** | |  |  |
|  | Albumin | 0.473 (0.450-0.497) | <.01 |
|  | Bilirubin | 1.061 (1.048-1.074) | <.01 |
|  | Uric acid | 1.064 (1.048-1.079) | <.01 |
|  | Hemoglobin | 0.928 (0.916-0.940) | <.01 |
|  | Total cholesterol | 1.000 (1.000-1.001) | .41 |
| **CT count** | |  |  |
|  | No CT | reference |  |
|  | >0~1/year | 1.471 (1.369-1.581) | <.01 |
|  | >1~3/year | 1.755 (1.632-1.888) | <.01 |
|  | ≥3/year | 2.834 (2.632-3.052) | <.01 |
| **Clinical trial** | | 1.734 (1.530-1.965) | <.01 |
| **Cancer treatment** | |  |  |
|  | Surgery only | reference |  |
|  | Surgery and chemotherapy | 2.091 (1.979-2.209) | <.01 |
|  | Chemotherapy only | 1.882 (1.775-1.997) | <.01 |
|  | No surgery and chemotherapy | 0.690 (0.651-0.733) | <.01 |

^a^Adjusted for sex, age, hypertension, diabetes, smoking, body mass index, mean arterial pressure, cancer type, eGFR at diagnosis, albumin, bilirubin, uric acid, hemoglobin, CT count, clinical trial and cancer treatment

*Abbreviations*: CBD, common bile duct; GB, gallbladder; eGFR, estimated glomerular filtration rate; CT, computed tomography
